# Peak Inhalation Exposure Metrics Used in Occupational Epidemiologic and Exposure Studies

**DOI:** 10.3389/fpubh.2020.611693

**Published:** 2021-01-08

**Authors:** M. Abbas Virji, Laura Kurth

**Affiliations:** Respiratory Health Division, National Institute for Occupational Safety and Health, Centers for Disease Control and Prevention, Morgantown, WV, United States

**Keywords:** peak metrics, epidemiologic studies, exposure assessment (EA), acute effects, chronic effects

## Abstract

Peak exposures are of concern because they can potentially overwhelm normal defense mechanisms and induce adverse health effects. Metrics of peak exposure have been used in epidemiologic and exposure studies, but consensus is lacking on its definition. The relevant characteristics of peak exposure are dependent upon exposure patterns, biokinetics of exposure, and disease mechanisms. The objective of this review was to summarize the use of peak metrics in epidemiologic and exposure studies. A comprehensive search of Medline, Embase, Web of Science, and NIOSHTIC-2 databases was conducted using keywords related to peak exposures. The retrieved references were reviewed and selected for indexing if they included a peak metric and met additional criteria. Information on health outcomes and peak exposure metrics was extracted from each reference. A total of 1,215 epidemiologic or exposure references were identified, of which 182 were indexed and summarized. For the 72 epidemiologic studies, the health outcomes most frequently evaluated were: chronic respiratory effects, cancer and acute respiratory symptoms. Exposures were frequently assessed using task-based and full-shift time-integrated methods, qualitative methods, and real-time instruments. Peak exposure summary metrics included the presence or absence of a peak event, highest exposure intensity and frequency greater than a target. Peak metrics in the 110 exposure studies most frequently included highest exposure intensity, average short-duration intensity, and graphical presentation of the real-time data (plots). This review provides a framework for considering biologically relevant peak exposure metrics for epidemiologic and exposure studies to help inform risk assessment and exposure mitigation.

## Introduction

Exposures vary considerably over time however, exposure dynamics are not consistently incorporated in epidemiologic exposure-response modeling; instead, summary metrics are used. Summary metrics ideally reflect the underlying biological processes linking exposure to dose and ultimately to the adverse health outcome (1, 2). More commonly, summary metrics used in epidemiologic studies are driven by the types of exposure measurements and data available and are therefore often surrogates of dose including qualitative and quantitative metrics of peak exposure. Peak exposures, i.e., high-intensity exposures over short-duration, are of concern because they can potentially overwhelm the capacity of normal biological defense mechanisms and induce adverse acute and chronic health effects (3).

Peak exposure metrics are often used in studies of acute respiratory effects (4) and some chronic disease outcomes (5). Acute or irritant effects of peak exposures are well-recognized and correspondingly, short-term exposure limits (STEL) or ceiling limits have been established for many substances to protect against the effects of intermittent high exposures (6). Peak exposure to ammonia or hydrogen chloride can result in mild but not trivial effects such as respiratory irritation, while peak exposure to hydrogen cyanide or hydrogen sulfide can cause serious acute effects which can be fatal (7). Epidemiologic studies have also observed associations of peak exposures with chronic conditions such as cancers or asthma, in which intense exposure over a short-duration during a relevant time window in the disease process surpasses a threshold, and initiates biological responses that subsequently result in adverse health outcomes (8, 9). Recent outbreaks of acute or accelerated silicosis and rapidly progressive pneumoconiosis associated with short-term high silica-content dust exposures in coal miners or engineered stone fabrication workers exemplify chronic effects of high exposures over short periods (10–12).

While the concept of peak exposure is well-recognized, there is little consensus in the literature on characterizing peak exposure, specifically identifying relevant peak exposure characteristics such as exposure intensity, duration, time interval between peaks, frequency of peaks, aggregation of peaks or absolute (exposures above a target value) vs. relative peaks (exposures above average of the series) (8, 13, 14). Since peak exposure metrics are not consistently defined or utilized, they may be overlooked as an exposure metric in epidemiological studies where average or cumulative exposure metrics are used instead, potentially leading to bias in estimates of exposure-response associations (15). Selecting an inappropriate summary metric results in a form of non-differential exposure misclassification not often discussed in epidemiologic studies (2), that likely includes both random and systematic errors. While non-differential exposure misclassification often leads to attenuation bias, i.e., bias toward the null in the measure of association (16), systematic errors can lead to bias toward or away from the null depending on the direction of the systematic error. For example in two separate studies, cumulative exposure, a commonly used summary metric in chronic disease epidemiology was not associated with toluene diisocyanate (TDI)-related occupational asthma or beryllium sensitization, but significant associations were observed with metrics of TDI peak exposure events and average or highest beryllium job exposures, respectively (17, 18). Thus, use of inappropriate summary metric may have led studies to miss important associations.

### Biological Considerations for Defining Peak Exposure

The relationship between exposure and adverse health outcomes is complex, varies over time, and is linked through two processes, exposure-burden (toxicokinetics) and burden-effect (pharmacodynamics) relationships (1, 2, 7). Inhaled exposure undergoes absorption, distribution, metabolism and excretion, with burden at the target tissue accumulating over a relevant period (dose) leading to repair processes and accumulation of damage at the cellular level which ultimately determines the health outcome (19, 20). The rate of accumulation at the target tissue can affect the type and the severity of the outcome, and is in part dependent upon exposure variability and physicochemical characteristics of the substance; a high dose rate may alter metabolism (e.g., via saturation), overload repair mechanisms and amplify response leading to non-linear (not proportional to exposure) effects (3). Peak exposures are important when exposures are variable, and this variability is transmitted to variability in burden through rapid kinetics and short half-times as in the case of irritant and acute respiratory effects, as well as for some chronic outcomes with non-linear effects; examples of non-linear effects include changes in uptake or susceptibility, synergism or antagonism, and allergies, all leading to amplification of response (6, 8). In the latter case, highest exposure, exposure excursions or upper percentiles of exposure distribution during a biologically-relevant time window may be more appropriate (3). The dose and the rate of accumulation at the target tissue can be estimated using toxicokinetic models to understand exposure kinetics and develop relevant exposure or dose metrics for epidemiologic studies (3, 6, 21). Smith and Kriebel propose that appropriate summary metrics of the time-varying exposure or dose can be selected from one of four simplified dose-effect process models obtained by combining the time course of effects (reversible vs. non-reversible) with whether the effect is proportional to dose (discrete vs. proportional); episodic asthma exacerbation is an example of a reversible, discrete disease process with a short etiologic time interval for which peak exposure is a relevant summary metric (20).

### Issues in Measuring Peak Exposure

Exposure assessment ideally includes measurement data, which are variable in time scales (e.g., minutes, years) with different implications for exposure assessment depending on the health outcome. Peak exposure measurements for acute effects may be collected in the time scale of minutes to hours over days to weeks, whereas for chronic effects, full-shift measurements and their distributions over months to years may be relevant, depending on the observation period of the study (8). There is limited guidance from standard setting agencies or health and safety associations on exposure assessment strategies to define, measure, or interpret peak exposure (22, 23). Additionally, the little guidance that exists on sampling duration to assess peak exposures for comparison to STELs often call for 15-min sampling or averaging times, an approach historically based on the limitations of exposure monitoring (3). Sampling duration determinations should ideally account for the kinetics of the substance and the capabilities of analytical methods or real-time instruments (7, 19).

Peaks can arise from normal process variation (regular peaks) or from process upset conditions and non-routine operations (irregular peaks) (24, 25). Regular peaks are more likely identified using exposure data from routine monitoring, whereas irregular peaks can be missed without continuous monitoring to account for unplanned events, non-routine operations, or less-frequently performed tasks. Irregular peaks are more frequently identified qualitatively through inquiries of events/activities in questionnaire surveys or during investigations of occupational illnesses or accidents. Quantitative peak metrics are ideally obtained from real-time measurements however, they are often unavailable. Additional considerations include autocorrelation among successive measurements (6, 7) which can amplify health effects (3), limitations of sampling and analytical methods for short-duration measurements and performance capabilities of real-time instruments.

A variety of approaches using different averaging times or methods have been used to define peak exposure (4, 26). For the most part, the definitions of peak exposure are not based on explicit biological mechanisms and the toxicokinetics of the substance is generally not reported. These metrics are most useful when they are correlated with the true, biologically-relevant peak measure. This literature review aims to summarize peak exposure metrics commonly used in epidemiologic and exposure studies to improve our understanding of peak exposures and the considerations for defining peaks such as health outcome of interest, disease mechanism, kinetics of substance and sampling, and analytical capabilities of methods and instruments.

## Methods

### Search Strategy

A comprehensive search of the published literature was conducted to identify potentially relevant literature using peak exposure metrics in epidemiological and exposure studies. An initial literature search was conducted in 2014 and updated in 2017 and 2019 utilizing multiple databases including Medline, Embase, and Web of Science to identify articles with the search keywords “environmental monitoring,” “maximum allowable concentration,” “occupational exposure,” “threshold limit values,” “peak exposure(s),” “short-term exposure,” “highest exposures,” “exposure-tails,” “spills,” “accidental exposures,” “acute exposures,” “exposure excursions,” “irregular exposures,” and “intermittent exposures.” The final list of keywords was drawn in part from how peaks were referenced in the published literature. Bibliographies of selected articles were also searched for relevant literature to include, as well as articles obtained from personal archives. Overall, 652 references retrieved from these searches were combined in an Endnote library and duplicate references were removed resulting in 588 references. We also searched NIOSHTIC-2, a bibliographic database of occupational safety and health publications supported by the National Institute for Occupational Safety and Health (NIOSH) using the search term “peak exposure.” The search in NIOSHTIC-2 identified 563 NIOSH-supported abstracts, reports and publications.

### Study Selection

Full text versions of the references were obtained and reviewed by two reviewers. The first reviewer excluded studies that were not available in English or did not meet inclusion criteria. References were excluded if they used the term “peak” in the wrong context, e.g., “peak” season, “peak” expiratory flow; evaluated the impact of an intervention or prevention strategy without a defined “peak”; studied a non-adult population; lacked detailed peak metric data; or had ≤5 subjects. Literature identified through NIOSHTIC-2 were reviewed using the above exclusion criteria. NIOSH Health Hazard Evaluation and Survey reports (27) were excluded. Included studies were categorized as either epidemiological or exposure studies.

A second reviewer examined the studies to define the scope of the review and made a final determination on study inclusion. For the epidemiologic studies, the review was focused on all health outcomes associated with occupational chemical or particulate exposures. For the exposure studies, the scope was broadened beyond occupational chemical or particulate exposures to also include exposure to noise, electromagnetic radiation, and environmental pollutants. The first reviewer then examined full text, published articles and reports, and selected articles for indexing if the concept of peak exposure was mentioned together with peak characteristics e.g., sampling strategy, duration, summary metric, described in data extraction section below; articles were excluded if they referred to highest exposure as “exposure peaks” but did not use a peak exposure metric in epidemiologic analysis. If a full text version was not available but all relevant information could be extracted from the abstract, then the reference was included. Conference abstracts identified in the search were also included if all relevant information could be extracted from the conference abstract. The combined search strategies resulted in 1,215 studies from which 182 relevant studies were indexed and summarized.

### Data Extraction and Summarization

Data were extracted from each reference to create a database summarizing the health outcome for epidemiological studies and specific exposure characteristics for all studies. For epidemiological studies, the database included information on epidemiologic study design, industry and occupation, health outcome measure and classification, method and frequency of measuring health outcome, the observation period of the health outcome, and details on peak exposure including the substance, sample type, sampling strategy, exposure assessment period and frequency, averaging time, sampling instrument, and peak exposure summary metric. For exposure studies, the database included only information on exposure characteristics noted above. The final step was to group all epidemiologic studies into groups of similar health outcomes, and the exposure studies into groups of classes of exposure.

## Results

After exclusions, 72 epidemiologic studies and 110 exposure studies were retained, and relevant details, including peak metrics and exposure characteristics, were extracted and reported in Supplementary Tables 1, 2, respectively.

### Epidemiologic Studies Summaries

Table 1 summarizes the 72 epidemiological studies that evaluated relationships between peak chemical or particulate exposures and health outcomes. Chronic respiratory effects were most frequently examined (28%) followed by various cancers (21%) and acute respiratory and irritation effects (17%). Most studies (82%) evaluated a chronic health outcome and ascertained health outcomes using questionnaire (61%) and/or physiological, functional, or biochemical testing (54%). Studies evaluated peak exposure to particulates, gases, metals, solvents, or other substances often using personal sampling methods and task-based (36%) or full-shift (33%) time-integrated monitoring, qualitative assessments (28%), or real-time monitoring (25%). The most common peak exposure summary metrics included “Yes/No event” to an event or measurements above a target (62%) and the highest measured exposures (33%).

**Table 1:** Characteristics of peak exposures metrics used in epidemiologic studies.

**Health outcome/study characteristics**	**Overall *N* (%)**	**Acute respiratory *N* (%)**	**Chronic respiratory *N* (%)**	**Immune sensitization *N* (%)**	**Pneumoconiosis *N* (%)**	**Cancer *N* (%)**	**Neurobehavioral *N* (%)**	**Cardiovascular *N* (%)**	**Biomarker *N* (%)**
*N* (%)	72 (100)	12 (17)	20 (28)	6 (8)	4 (6)	15 (21)	9 (13)	3 (4)	3 (4)
**Epidemiologic study type**									
Intensive and follow-up	10 (14)	5 (42)	–	–	–	–	2 (22)	2 (67)	1 (33)
Cohort	25 (35)	–	11 (55)	–	3 (75)	9 (60)	1 (11)	1 (33)	–
Cross–sectional	23 (32)	4 (33)	7 (35)	6 (100)	–	–	5 (56)	–	1 (33)
Case–control	8 (11)	–	1 (5)	–	1 (25)	6 (40)	–	–	–
Case–series	2 (3)	–	1 (5)	–	–	–	1 (11)	–	–
Ecological	4 (5)	3 (25)	–	–	–	–	–	–	1 (33)
**Health outcome classification**									
Acute	22 (31)	12 (100)	2 (10)	–	–	–	4 (44)	2 (67)	2 (67)
Chronic	59 (82)	4 (33)	18 (90)	6 (100)	4 (100)	15 (100)	8 (89)	1 (33)	3 (100)
**Health assessment method**									
Questionnaire	44 (61)	10 (83)	19 (95)	5 (83)	–	–	9 (100)	1 (33)	–
Physical exam	4 (6)	–	–	–	–	–	4 (44)	–	–
Physiologic/functional or biochemical test	39 (54)	8 (67)	11 (55)	6 (100)	4 (100)	–	5 (56)	2 (67)	3 (100)
Death certificates/insurance	11 (15)	–	–	–	–	10 (67)	–	1 (33)	–
Registries/records	5 (7)	–	–	–	–	5 (33)	–	–	–
**Health observation period**									
Cross-shift	17 (24)	12 (100)	–	–	–	–	3 (33)	1 (33)	1 (33)
Cross-week	5 (7)	5 (42)	–	–	–	–	–	–	–
Over month(s)	7 (10)	2 (17)	2 (10)	–	–	–	1 (11)	–	2 (67)
Over years	52 (72)	2 (17)	18 (90)	6 (100)	4 (100)	15 (100)	5 (56)	2 (67)	–
**Exposure substance**									
Particulate	13 (18)	8 (67)	4 (20)	–	–	–	–	1 (33)	–
VOC	2 (3)	2 (16)	–	–	–	–	–	–	–
Isocyanates (HDI, TDI, MDI)	4 (6)	1 (8)	3 (15)	–	–	–	–	–	–
Gases	9 (13)	–	6 (30)	–	–	–	1 (11)	1 (33)	1 (33)
Diacetyl	2 (3)	–	2 (10)	–	–	–	–	–	–
Irritants and allergens/JEM	2 (3)	–	2 (10)	–	–	–	–	–	–
Other chemicals	5 (7)	–	2 (10)	2 (33)	–	–	–	1 (33)	–
Silica	4 (6)	–	–	–	3 (75)	1 (7)	–	–	–
Asbestos	2 (3)	–	–	–	1 (25)	1 (7)	–	–	–
Metals	8 (11)	–	1 (5)	4 (66)	–	–	3 (33)	–	–
Solvents	8 (11)	–	–	–	–	3 (20)	5 (56)	–	–
Benzene	5 (7)	–	–	–	–	5 (33)	–	–	–
Formaldehyde	7 (10)	1 (8)	–	–	–	5 (33)	–	–	1 (33)
Pesticides	1 (1)	–	–	–	–	–	–	–	1 (33)
**Sampling strategy**									
Real-time	19 (26)	10 (83)	4 (20)	–	–	1 (7)	2 (22)	1 (33)	1 (33)
Task time integrated	26 (36)	5 (42)	5 (25)	5 (83)	–	7 (47)	2 (22)	1 (33)	1 (33)
Full shift time integrated	24 (33)	4 (33)	6 (30)	4 (67)	–	7 (47)	2 (22)	–	1 (33)
Modeling	2 (3)	1 (8)	–	–	1 (25)	–	–	–	–
Qualitative/Judgment/ JEM	20 (28)	1 (8)	13 (65)	–	3 (75)	5 (33)	2 (22)	1 (33)	–
Biomarkers	3 (4)	–	–	–	–	–	3 (33)	–	–
**Sample type**									
Personal	33 (46)	10 (83)	11 (55)	3 (50)	–	–	6 (67)	2 (67)	1 (33)
Area	16 (22)	3 (25)	4 (20)	5 (83)	–	–	2 (22)	–	–
**Sample averaging time**									
Instantaneous/ instrument	7 (10)	4 (33)	2 (10)	–	–	–	1 (11)	–	–
Short-duration (15 min.)	19 (26)	7 (58)	–	–	–	8 (53)	2 (22)	1 (33)	1 (33)
Variable task duration (0.5–4 h)	11 (15)	3 (25)	4 (20)	3 (50)	–	–	–	1 (33)	–
Full-shift/session (4–8 h)	16 (22)	1 (1)	6 (30)	4 (67)	–	3 (20)	2 (22)	–	–
**Peak exposure summary metric**									
Average intensity short-term	16 (22)	7 (58)	–	2 (33)	–	4 (27)	2 (22)	–	1 (33)
Highest intensity/category	24 (33)	4 (33)	5 (25)	4 (67)	1 (25)	5 (33)	2 (22)	2 (67)	1 (33)
Duration > target	5 (7)	2 (17)	–	1 (17)	–	2 (13)	–	–	–
Frequency #/% > target	17 (24)	4 (33)	2 (10)	1 (17)	–	8 (53)	2 (22)	–	–
Yes/no event, > target	27 (38)	1 (8)	14 (70)	3 (50)	3 (75)	2 (13)	3 (33)	–	1 (33)
95th percentile	2 (3)	–	1 (5)	1 (17)	–	–	–	–	–
Variability (GSD)	1 (1)	–	–	1 (17)	–	–	–	–	–

*Note: percentages can be >100 when multiple characteristics are present in one study, or <100 when looking at only a sub portion of the data*.

Of the 12 studies evaluating acute respiratory and irritation effects (4, 28–38), all studies evaluated cross-shift acute effects while four studies also evaluated chronic effects by follow-up assessments. Questionnaires were commonly used to assess health outcomes (83%), while spirometry or biochemical tests were conducted in 67% of studies. Most studies (67%) evaluated exposures to particulate matter including dust, welding fumes, wood dust, nano materials, and sodium borate, using personal monitoring (83%) and real-time instruments (83%). For averaging times, 92% of studies utilized 15-min averages or instantaneous measurements (recording interval depended on the instrument). Peak metrics used included average short-duration intensity (58%), highest intensity (33%), and frequency greater than a target concentration (33%).

Most of the 20 studies evaluating chronic respiratory effects (39–58) were cohort studies (55%) focused on asthma (55%), and two also evaluated acute health effects. Questionnaires were used to assess health outcomes in most studies (95%) along with spirometry or biochemical tests (55%). Asthma, respiratory symptoms, and other acute respiratory diseases were most frequently inferred through questionnaire or self-reports. Studies most frequently (30%) evaluated exposure to gases (ozone, sulfur dioxide, and chlorine), and the remainder included particulates (soy dust, metal dust, rayon, and nylon flock dust), isocyanates, diacetyl, other chemicals, and irritants and allergens from a job-exposure matrix (JEM). Qualitative assessment of peak events (65%) was common, as was personal monitoring (55%) using full-shift (30%) sampling. Peak metrics used included Yes/No event (70%) and highest intensity (25%). Some studies collected real-time particulate measurements (42, 44, 45) or real-time or time-integrated diacetyl measurements (51, 52) to characterize peak exposures during specific tasks, but used qualitative metrics indicating the performance or frequency of a task in the analysis.

All six sensitization studies (18, 59–63) were cross-sectional and evaluated sensitization from beryllium (67%) and hexahydrophthalic anhydride or methylhexahydrophthalic anhydride (33%) via serological testing. All studies conducted either personal (50%) or area monitoring (83%), using full-shift sampling (67%) or short duration task sampling (83%); real-time instruments were not used in any of the studies. Frequently reported peak metrics included highest intensity (67%) and Yes/No event (50%).

The four pneumoconioses studies (8, 64–66) evaluated silicosis and pneumoconiosis from silica and dust exposures (75%) and pleural plaques from asbestos exposure (25%) via radiographic methods. Some exposure monitoring was conducted to create qualitative peak metrics but was not directly used in epidemiologic studies. Peak metrics used included Yes/No event (75%) and highest intensity (25%).

Of the 15 studies evaluating cancer (67–81), most (67%) were mortality studies from death certificates and insurance records, and 33% identified cases from cancer registries and medical records. Studies evaluated exposures to formaldehyde, benzene, silica, asbestos, total hydrocarbons, styrene, and acrylonitrile. The most common exposure assessment methods were short duration task and full-shift monitoring (47% each) and 53% of studies had averaging times of 15 min or less. The most used summary metric was frequency greater than a target concentration (53%) and highest intensity (33%). Several studies (67, 69, 70, 79, 81) did not report details of exposure assessment methods e.g., measurement type or type of sampling. Most studies examined multiple peak exposure metrics.

Of the nine neurobehavioral studies (82–90), eight studies (89%) evaluated a chronic outcome, while four studies (44%) also evaluated acute effects. All studies assessed health effects by questionnaire and a combination of clinical evaluation and physiological or functional testing. Exposures most frequently evaluated included organic solvents (vinyl chloride, trichloro ethylene, styrene, etc., *n* = 5), while three studies (33%) evaluated metals exposures (mercury, lead). Personal monitoring was conducted in 67% of studies and over half (55%) reported 15-min averages or full-shift exposures. A variety of summary peak exposure metrics were used including Yes/No event (33%), yet several studies (82, 84) did not utilize the exposure metrics in epidemiological analysis.

Of the three studies on cardiovascular outcomes (91–93), two were panel studies and one was a historical cohort evaluating cardiovascular mortality from death certificates. Substances evaluated included PM_2.5_, carbon disulfide and dioxin. Two studies conducted personal exposure monitoring utilizing 24-h real-time monitoring and short duration sampling. Peak metrics included highest intensity (67%) and Yes/No event (33%).

Of the three studies assessing biomarkers (94–96), all were assessed via physiologic or biochemical testing. Substances evaluated included pesticides, carbon monoxide, and formaldehyde. Two studies conducted exposure monitoring, one utilizing real-time monitoring and one short-duration/full-shift sampling. Peak metrics included highest intensity (33%), average short duration (33%), and Yes/No event (33%).

### Exposure Studies Summaries

Table 2 summarizes the 110 studies that evaluated a peak exposure to a chemical, particulate, or physical exposure in occupational or environmental settings. Exposures evaluated included environmental exposures (31%), organic chemicals (28%), particulates (22%), gases (12%), and physical agents (12%). Exposure studies most frequently assessed exposure by personal monitoring sampling (66%), using real-time sampling strategies (76%), and short-duration sample averaging time (24%). Averaging times varied and highest exposure intensity (93%) was the most frequently reported peak exposure summary metric.

**Table 2:** Characteristics of peak exposures metrics described in exposure studies.

**Study characteristic**	**Overall**	**Particulate matter**	**VOC/solvents**	**Gases**	**Physical agents**	**Environmental pollutants**
	*N* (%)	*N* (%)	*N* (%)	*N* (%)	*N* (%)	*N* (%)
*N* (%)	110 (100)	24 (22)	31 (28)	13 (12)	13 (12)	34 (31)
**Sampling strategy**						
Real-time	84 (76)	20 (83)	13 (42)	12 (92)	13 (100)	26 (76)
Grab-instantaneous	4 (4)	–	3 (10)	–	–	1 (3)
Task time integrated	26 (24)	4 (17)	17 (55)	1 (8)	–	4 (12)
Full shift time integrated	7 (6)	2 (8)	1 (3)	–	–	4 (12)
Modeling	3 (3)	–	2 (6)	–	–	1 (3)
Qualitative/Judgment/JEM	3 (3)	–	2 (6)	–	–	–
**Sample type**						
Personal	73 (66)	15 (63)	17 (55)	10 (77)	11 (85)	20 (59)
Area	50 (45)	13 (54)	14 (45)	5 (38)	4 (31)	14 (41)
NA or Not reported	9 (8)	–	7 (23)	–	–	1 (3)
**Sample averaging time**						
Instantaneous/instrument	53 (48)	14 (58)	13 (42)	9 (69)	5 (38)	12 (35)
Short-duration (15 min.)	18 (16)	2 (8)	12 (39)	3 (23)	–	1 (3)
Variable task (0.5–4 h)	47 (43)	7 (29)	11 (35)	5 (38)	6 (46)	18 (53)
Full-shift/session (4–8 h)	18 (16)	1 (4)	1 (3)	–	6 (46)	10 (29)
Not reported	5 (5)	1 (4)	2 (6)	–	–	1 (3)
**Peak exposure summary metric**						
Highest intensity/category	102 (93)	21 (88)	27 (87)	12 (92)	12 (92)	30 (88)
Plots	35 (32)	11 (46)	6 (19)	6 (46)	–	12 (35)
Average intensity short-term	51 (46)	11 (46)	21 (68)	5 (38)	5 (38)	9 (26)
95th percentile	21 (19)	5 (21)	5 (16)	1 (8)	3 (23)	7 (21)
Duration > target	10 (9)	2 (8)	2 (6)	1 (8)	2 (15)	2 (6)
Frequency #/% > target	24 (22)	4 (17)	7 (23)	3(23)	5 (38)	5 (15)
Yes/No event, > target	2 (2)	1 (4)	–	–	–	–
Variability (GSD)	7 (6)	3 (13)	2 (6)	–	2 (15)	–
Other	7 (6)	1 (4)	1 (3)	1 (8)	3 (23)	1 (3)

*Percentages can be >100 when multiple characteristics are present in one study, or <100 when looking at only a sub portion of the data*.

The 24 (22%) studies measuring peak particulate matter exposure (14, 26, 97–118) included assessments for fine, ultrafine, and nanoscale particulates (50%), respirable, inhalable or total dust (38%), or specific substances (21%) (e.g., beryllium, black carbon). For quantitative exposure, 63% conducted personal monitoring mostly using real-time instruments (83%). Over half (58%) reported instantaneous measurements and 29% reported variable short-duration average intensity. The most frequently reported peak metrics included highest intensity (88%), plots (46%), and average short-duration intensity (46%).

The 31 (28%) studies measuring peak exposure to organic chemicals (13, 116, 119–147) included assessments for volatile organic compounds (VOCs) (29%), solvents (32%), formaldehyde (16%), and other hydrocarbons (19%). Personal monitoring was conducted in 55% of studies using short-duration (55%) and real-time monitoring (42%). Time intervals for sampling included instantaneous measurements (42%), 15-min (39%) and variable short-duration (25%) average exposures, and peak metrics included highest intensity (87%) and average short-duration intensity (68%).

The 13 (12%) studies measuring peak exposure to gases (104, 108, 119, 123, 148–155) included assessments for carbon monoxide (46%), oxides of nitrogen (31%), carbon dioxide and ammonia (23% each), hydrogen sulfide (15%), and sulfur dioxide and chlorine (8% each). Personal monitoring was conducted in 77% of studies most frequently utilizing real-time instruments (92%) and instantaneous measurements (69%). Peak metrics used most frequently included highest intensity (92%), plots (46%), and average short-duration intensity (38%).

The 13 (12%) studies measuring peak exposure to physical agents (110, 156–167) included assessments for noise (46%) and electromagnetic frequency (54%). Personal monitoring was conducted in 85% of studies, all utilizing real-time instruments. Studies reported variable short-duration average intensity (46%), full-shift measurements (46%), and instantaneous measurements (38%). Most frequently reported peak metrics included highest intensity (92%), average short-duration intensity (38%), and frequency greater than a target concentration (38%).

One occupational study (168) developed a qualitative (Yes/No event) peak accidental exposure metric based on professional judgment for a JEM, which did not fit into any of the above exposure categories and was not included in Table 2.

Environmental exposure studies reported a variety of peak exposure metrics. The 34 (31%) studies measuring peak exposure to environmental pollutants (125, 169–201) included assessments for particulate matter (53%), polycyclic aromatic hydrocarbons (PAH)/VOCs (21%), oxides of nitrogen, carbon monoxide/dioxide and black carbon (12% each), ozone (6%) and ammonia, metal and noise (3% each). Personal monitoring was conducted in 59% of studies most often utilizing real-time instruments (76%) and short-duration monitoring (12%). Most studies (53%) reported variable short-duration average intensity or instantaneous measurements (35%). The most reported peak metrics included highest intensity (88%) and plots (35%).

## Discussion

A comprehensive literature review was conducted to summarize peak exposures in epidemiologic and exposure studies in order to better understand peak exposures and instill consistency in measuring and creating peak metrics for future studies. Most epidemiologic studies evaluated a chronic effect, and fewer considered acute effects for which peak or short-duration exposures would be relevant and advantageous, and for which STELs are more often specified. Interestingly, quantitative exposure data were available for most studies, but were not of adequate quality or detail to construct more complex metrics beyond binary, maximum or average exposure metrics. Most studies did not explicitly note the disease mechanism or biological basis for the postulated peak exposure-response relationships which are necessary to construct biologically valid peak exposure summary metrics to ensure valid health risk estimates (9, 20). Future studies could benefit from multi-disciplinary collaborations in developing novel metrics of peak exposure and to better capture the biological processes underpinning the exposure-response relationships.

The expanded search of exposure studies to include physical hazards and environmental pollutants provided a broader context of peak characterization. Most exposure studies utilized real-time or short-duration task-based sampling with sampling duration ≤15 min. Highest exposure intensity was the most frequently reported peak exposure summary metric, but average short-duration intensity and plots were also common. Statistical methods that account for non-stationary autocorrelation or measurements below the detection limit in real-time exposure data were not considered, instead favoring examples of plots and highest measured exposure. Peak metrics can be used in epidemiologic studies, or in industrial hygiene applications to make decisions on interventions, or to evaluate the efficacy of control measures (112). Peak exposures during specific tasks can have a substantial impact on full-shift average exposures, and this information can be used to design efficient control strategies focused on limited high-exposure tasks (14) and to demonstrate the benefits of changing work practices when linked to video exposure monitoring.

Epidemiologic studies of acute effects were predominantly for respiratory outcomes utilizing personal real-time exposure measurements summarized over 15 min or less. The most common peak exposure summary metrics were average and highest short-duration exposure, as well as the frequency and duration of measurements above a target value. Even within this narrower class of health outcomes focused on acute respiratory effects, there was little consistency in exposure data used or summary metrics created, and little discussion on biological basis for defining peak exposure and the underlying biological processes linking exposure to health outcome. Notable exceptions include a set of studies on the acute respiratory effects of sodium borate dust which measured real-time exposures of 106 participants on 4 consecutive days and real-time self-reported symptoms and their severity, as well as symptoms and spirometry at hourly interval during work hours (4, 26, 29, 37, 202). These studies used 15-min average exposure and nasal dose estimates and explained their choice of averaging time in terms of the underlying biological processes and the time course of experiencing respiratory irritation (29, 38). From the review of exposure studies, novel and interesting metrics of peak exposures based on intensity, duration, time interval between peaks, frequency, and aggregation of peaks, and absolute vs. relative peaks from real-time data have been proposed but not utilized in epidemiologic studies. A study of healthcare workers utilized a Bayesian approach to model VOC exposure time-series to estimate 5- and 15-min, task-specific exposure summaries including (posterior distributions of) geometric means (GM), geometric standard deviations (GSD), and quantiles such as 95th percentile (P95) (147). In another study of electric utility workers exposed to extremely low-frequency magnetic fields, exposure patterns were described by conducting a frequency-domain time-series analysis and grouping workers based on the frequency patterns of exposure time series (203). Studies of VOC exposures in spray painting operators (13) and dust exposures in flour processing workers (14), created a range of relative or absolute peaks using 5-s, 1- or 15-min moving averages, including: number of peaks per hour, duration of peaks, average, and maximum concentrations of the peaks and their ratios, and average duration between peaks. Understanding the effect of short-duration exposures, there are efforts to develop task or job-task exposure matrix for use in epidemiological studies (204, 205).

Epidemiologic studies of chronic effects evaluated various health outcomes including respiratory, cancer, cardiovascular and neurobehavioral outcomes. Quantitative measurements were available for many studies, however, most used peak exposure summary metrics including Yes/No to an event and the highest or average intensity. Notably, a study of beryllium manufacturing workers created and evaluated a range of qualitative and quantitative peak exposure metrics including summaries (GM, GSD, Maximum, P95, frequency > target, duration of exposure, duration > target) from full-shift and short-duration exposure measurements; qualitative metrics included company recorded events such as leaks and upset conditions, ventilation or equipment failure and reportable spills, and instances of evacuations, and self-reported high exposures, and participation in decontamination and spills clean-ups, to assess their utility in predicting beryllium sensitization (61). This study reported the biological processes underpinning the relationship between peak exposure and sensitization and found moderate to high correlation among the various metrics with most performing equivalently in epidemiological analyses. Notably, the reviews by Checkoway et al. (8, 9) highlight the challenges of assessing peak exposures for chronic disease where the timing of exposure is often important and detailed information on peak exposures over a period of years is often lacking.

Of the 72 epidemiologic studies, 52 evaluated multiple metrics in their study, such as peak, average or cumulative (Supplementary Table 1). Of these studies, many did not observe significant associations between the health outcome and any of the exposure metrics or did not report all measures of association. However, in 10 studies, peak metrics were reported to perform better than average or cumulative exposure metrics in terms of the magnitude of effect, albeit not always statistically or clinically significant. In these studies, exposure-response relationships based on accepted biological plausibility often demonstrated significant associations with peak metrics (44, 64, 66, 69, 75, 87). For example, risk of silicosis was better predicted by brief, short-term quartz exposure (64) and relative peak quartz exposures (as well as average non-peak exposures) compared to cumulative exposures (69). Likewise, high dust levels from peak quartz measures were a better predictor of pneumoconiosis than average exposures in a study of iron ore mine and beneficiation plant workers (66). Many studies evaluating multiple exposure metrics reported moderate to high correlations between average and peak exposure metrics that often resulted in either an association with all exposure metrics or no association with any exposure metrics. Ultimately, consideration of biological processes that underlie the exposure-response relationship in creating or selecting exposure metrics will lead to a more systematic and justified approach to defining peak metrics, though not necessarily a unified definition of peaks.

The often-used time-integrated sampling methods, while specific, are not time-resolved and do not capture peaks, requiring use of peak surrogates. There is renewed and growing interest in the development of direct reading instruments that have improved sensitivity, detection limit, specificity, multiplexing capability, and other enhanced performance characteristics (206), which hold tremendous promise of novel peak exposure metrics for use in epidemiologic studies of acute effects (13, 14, 203, 207). Real-time exposure monitoring provides flexibility in *post-hoc* definitions of peaks, and in examining the correlations among the various peak metrics and their utility in predicting risk of health effects. To emphasize the importance of developing and using direct reading real-time instrumentation in exposure assessment, NIOSH launched the direct reading exposure assessment methods (DREAM) initiative and established the NIOSH Center for Direct Reading and Sensor Technologies (NCDRST) to coordinate research and develop guidance on direct reading and sensor technologies. Despite the historical use and renewed interest in direct reading instruments for exposure assessment, there is limited quantitative exposure metrics generated by these instruments for use in occupational exposure assessment and epidemiology. As shown in this review, while real-time data are often used in exposure and epidemiologic studies, they are not appropriately summarized or fully utilized, often resorting to calculating averages, selecting highest exposures, or displaying plots of typical profiles. Some studies have provided guidance on extracting various metrics of peak exposure in the time or frequency domains of real-time data based on intensity, duration, time interval between peaks, frequency, aggregation of peaks, and patterns based on the frequencies at which high amplitude (peaks) occur (13, 14, 203). Principal components analysis of these peak metrics revealed three independent factors related to intensity, variability, and duration that sufficiently characterized peak exposures (13), which is consistent with their expression in terms of real-time exposure distribution parameters and autocorrelation, i.e., the GM, GSD, and autocorrelation coefficient (207). Recently, a statistically rigorous method was proposed to analyze real-time data that simultaneously accounts for censored data, fixed-effects covariates, hierarchical random-effects, and non-stationary autocorrelation and was fit using Markov-Chain Monte Carlo within a Bayesian context; the model provides a range of summary measures such as the mean, standard deviation and various quantiles of interest for a choice of short-durations e.g., 15-min (208). This method was used in a study of healthcare workers' exposure to VOCs in which peaks were quantified as the median of the posterior distributions of the 95th percentile of short-duration (15-min averages) task, a metric that is easy to interpret when used in epidemiologic studies, to make decisions on interventions, or to evaluate the efficacy of control measures (147). While limited guidance and examples exist on analyzing real-time data, these methods are complex, requiring familiarity with advanced statistical methods and packages making their wide-spread use unlikely. More user-friendly tools are needed to encourage more regular use and application of such complex methods to analyze real-time data.

Certain limitations exist in this study. While a comprehensive literature search was conducted involving searching multiple databases and references of identified studies to protect against selection bias, the search was not exhaustive. In addition, we found peak metrics to be highly diverse and inconsistent across studies resulting in challenges summarizing the reviewed studies and deriving generalizable definition of peaks and performance of various peak metrics. Only one reviewer selected studies for the review; two independent reviewers may have decreased the chance of systemic error and bias. The search terms were designed to be more inclusive of studies, especially given the variety of terms used to describe the concept of peak exposure; the search also included abstracts with sufficient information on the peak exposure metrics to complete the data extraction table (Supplementary Tables 1, 2) e.g., sampling strategy, duration, summary metric. However, studies of short-duration task exposures that did not address the concept of peak exposures were excluded. Studies were included without consideration of the magnitude or statistical significance of association, as the objective was to summarize the metrics used and not the strength of any particular exposure-response relationship. Restricting this review to epidemiologic studies of chemical and particulate peak exposures kept the scope of the review manageable but limited its comprehensiveness as peak exposures to physical and environmental hazards in epidemiological studies were not assessed.

## Conclusion

Peak or short-duration exposures are important in studying acute effects as well as some chronic conditions, but peaks are not consistently defined or used in epidemiologic studies and limited guidance exists on strategies for monitoring peak exposures. This review provides an overview of peak exposure metrics commonly used in epidemiologic and exposure studies and identifies the challenges of conducting exposure assessments. While consensus recommendations are not provided or a single peak metric is not recommended, examples of peak exposure approaches and considerations for defining peak are provided. Exposure assessment for epidemiologic studies requires consideration of the complex biological processes underlying exposure-response relationships, which are often unknown. Numerous factors determine the assessment of peak exposures including exposure patterns and variability, physicochemical properties of the exposure substance, the disease mechanism and time course, as well as exposure levels at which effects occur. This review summarizes peak exposure metrics, highlights examples of studies, and identifies factors to consider in developing peak exposure metrics for epidemiologic exposure-response studies that will result in accurate estimates of health risks and appropriate exposure mitigation strategies to reduce morbidity and mortality.

## Author Contributions

MV and LK contributed to all stages of the manuscript from conception and design of the literature search to review of selected papers, extraction summarization of papers, drafted, revised, and approved the submitted version of the manuscript. All authors contributed to the article and approved the submitted version.

## Conflict of Interest

The authors declare that the research was conducted in the absence of any commercial or financial relationships that could be construed as a potential conflict of interest.
